# Induction of plasticity in the human motor cortex by pairing an auditory stimulus with TMS

**DOI:** 10.3389/fnhum.2014.00398

**Published:** 2014-06-03

**Authors:** Paul F. Sowman, Søren S. Dueholm, Jesper H. Rasmussen, Natalie Mrachacz-Kersting

**Affiliations:** ^1^Department of Cognitive Science, Macquarie UniversitySydney, NSW, Australia; ^2^Perception and Action Research Centre (PARC), Faculty of Human Sciences, Macquarie UniversitySydney, NSW, Australia; ^3^Australian Research Council Centre of Excellence in Cognition and its Disorders (CCD), Macquarie UniversitySydney, NSW, Australia; ^4^Department of Health Science and Technology, Center for Sensory-Motor Interaction (SMI), Aalborg UniversityAalborg, Denmark

**Keywords:** paired associative stimulation, transcranial magnetic stimulation, auditory motor integration, speech sounds, plasticity, motor cortex, auditory cortex

## Abstract

Acoustic stimuli can cause a transient increase in the excitability of the motor cortex. The current study leverages this phenomenon to develop a method for testing the integrity of auditorimotor integration and the capacity for auditorimotor plasticity. We demonstrate that appropriately timed transcranial magnetic stimulation (TMS) of the hand area, paired with auditorily mediated excitation of the motor cortex, induces an enhancement of motor cortex excitability that lasts beyond the time of stimulation. This result demonstrates for the first time that paired associative stimulation (PAS)-induced plasticity within the motor cortex is applicable with auditory stimuli. We propose that the method developed here might provide a useful tool for future studies that measure auditory-motor connectivity in communication disorders.

## Introduction

Paired associative stimulation (PAS) is a technique used to experimentally induce long-lasting changes in cortical excitability (Stefan et al., 2002; Ridding and Flavel, 2006; Mrachacz-Kersting et al., 2007; Murakami et al., 2008; Kumpulainen et al., 2012). Most commonly in PAS studies, electrical stimulation of the median nerve is paired with transcranial magnetic stimulation (TMS) of the contralateral motor cortex. The nerve impulse resulting from the somatosensory stimulus can be timed to arrive at the cortical level milliseconds prior the TMS pulse in order to induce a long-lasting increase in excitability—a process that is thought to be mediated by a Hebbian long-term potentiation (LTP)-like process (Stefan et al., 2002).

In recent years, modified PAS protocols have been designed that apply more ecologically valid stimuli in place of either the TMS or the electrical somatosensory stimulation, e.g., TMS paired with movement (Thabit et al., 2010) or electrical somatosensory stimulation paired with motor imagery (Mrachacz-Kersting et al., 2012). PAS protocols have also moved beyond ubiquitous sensorimotor associations to demonstrate that pairing a TMS-induced cortical activation outside the motor cortex with a homotopic sensory activation can induce enhanced responses to sensory inputs. For example, Schecklmann et al. (2011) showed that pairing a TMS pulse to the auditory cortex with a simple tone could induce a prolonged decrement of the auditory evoked potential. Cortical stimulation has also been paired with visual stimuli to demonstrate the capacity for visuomotor integration to mediate plastic changes in motor cortex (Suppa et al., 2013). To date however, the connections known to exist between the auditory and motor domains have not been tested for their capacity to induce motor cortex plasticity.

A number of well-described functional links between audition and the motor system exist. These range from protective reflexive motor activations in response to signals of potential danger (Forbes and Sherrington, 1914) to the complex feedback and feedforward communication necessary for fluent speech to occur (Tourville et al., 2008; Perkell, 2012). These connections allow us to, for example, modulate the volume of our speech to appropriately match the ambient environmental noise (Lane and Tranel, 1971) or modulate the sensitivity of our sensory system to compensate for speech-induced reafference (Curio et al., 2000).

Motoric activation via auditory inputs has been demonstrated in a number of experiments that have used TMS to probe the link between speech perception and motor representations. Modulation of motor cortical excitability during speech perception has been demonstrated to occur in the cortical representations of the hand (Flöel et al., 2003), lips (Watkins et al., 2003) and tongue (Fadiga et al., 2002; Roy et al., 2008).

Despite evidence suggesting a strong connection between auditory and motor centers, auditory stimuli have not yet been used in a modified PAS study to induce plasticity in the motor area. The aim of the current study was to investigate whether it is possible to induce plasticity in the motor system by pairing auditory stimuli and TMS. The development of such a protocol would in future allow for the direct investigation of auditorimotor linkages in a number of disorders where these are thought to be abnormal. Auditorimotor disconnection or dysfunction has, for example, been proposed to underpin the speech dysfluencies that characterize stuttering (Neef et al., 2011) and the misattribution of self-produced speech that may produce auditory hallucinations in schizophrenia (Ford et al., 2005).

## Methods

Two separate experiments (A and B) were conducted. Given that the timing of stimuli in a PAS protocol is critical for facilitating plastic change (Stefan et al., 2002; Wolters et al., 2005; Mrachacz-Kersting et al., 2007; Murakami et al., 2008; Kumpulainen et al., 2012), the aim of Experiment A was to find, at a group level, the optimal offset timing of the motor cortical excitation from the onset of the auditory stimulus. This offset was determined by applying TMS pulses at different latencies relative to the onset of the auditory stimulus and measuring the conditioned motor evoked potential (MEP) in the right first dorsal interosseus (FDI) muscle.

The PAS protocol implemented in Experiment B was informed by the results of Experiment A. First a baseline session was conducted where MEPs (TMS with no auditory stimuli) were collected and saved as pre-PAS measurements. This was followed by an intervention block which consisted of the auditorimotor PAS-protocol. During the intervention block, subjects received an auditory stimulus paired with TMS using the optimal time latency between stimulations that was found in Experiment A. After the intervention session, post-PAS MEPs were recorded immediately after and then 15 min after the session ended (post and post15, respectively). By comparing pre- with post-MEPs and post15-MEPs it was possible to evaluate whether motor cortex excitability changes had occurred, how fast they evolved and whether they were long-lasting.

## Experiment A—timing of stimuli

### Subjects

Experiment A was performed on 12 healthy right-handed volunteers (9 males), aged 18–36 years (mean 24.2 ± 5.0 years). Prior to commencement of the experiment subjects completed a standard TMS screening questionnaire and provided written informed consent. None of the subjects reported any history of hearing impairment, neurological disease or mental illness, was taking regular medication or had a history brain injury. This study was reviewed and approved by the Human Research Ethics Committee of Macquarie University.

### Experimental procedure

Subjects were seated in a chair with their right arm and hand resting in a comfortable position on an armrest. An armrest was used in order to eliminate hand movements during recordings. During the experiment the subject was told to relax, avoid any movement of the right arm and hand and to have their eyes open. Surface EMG (sEMG) was recorded (1000 s× gain, bandpass filtered from 20–500 Hz) from a bipolar electrode (Medi-Trace 100, Kendall/Tyco Healthcare, USA) montage. One electrode was placed over the muscle belly of the right FDI muscle and the other electrode was placed over the proximal metacarpal of the index finger.

A monophasic transcranial magnetic stimulator (Magstim model 200, Magstim, Whitland, UK), with a focal figure-of-eight stimulating coil (90-mm outer diameter), was used to elicit MEPs from the right FDI muscle. The stimulating coil was held tangentially to the skull with the coil oriented 45° to the parasagittal plane and the handle pointing laterally and posteriorly. The center of the coil junction was placed over the primary motor cortex (M1) hand area of the left hemisphere and the “motor hot spot” was determined as the site where TMS consistently elicited the largest MEPs.

**Figure 1:**
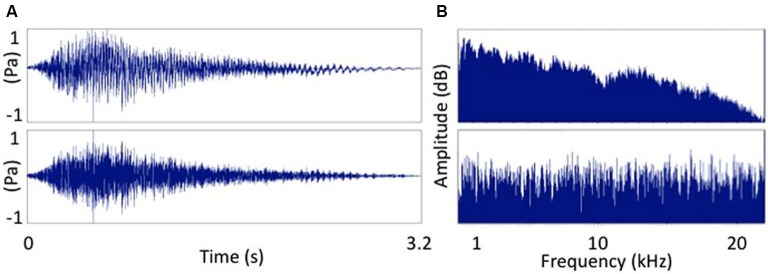
**Two sounds used as auditory stimuli. (A)** The word “Hey!” and **(B)** signal correlated noise version of **(A)**. Frequency spectra of the two auditory stimuli. **(A)** The word “Hey!” and **(B)** signal correlated noise (white noise) version of **(A)**.

Resting motor threshold (MT) was determined by finding the lowest stimulation intensity of the motor hotspot for the right FDI needed in order to obtain an MEP with a peak-to-peak amplitude of 50 µV in 5 out of 10 consecutive stimulations. The TMS test intensity was then set at 120% of resting MT. Eight different TMS conditions were tested. These consisted of seven auditory-stimulation/TMS pairs and one TMS condition without associated auditory stimulation (baseline). The auditory-stimulation/TMS pairs consisted of a test TMS pulse applied at one of seven different intervals (25, 50, 100, 150, 200, 250 and 300 ms) after the onset of the auditory stimulus. The auditory stimulus consisted of a male voice pronouncing the word “Hey!” played back at 80 dB SPL via Etymotic ER-1 insert tube-phones. We chose to use a speech sounds because previous research suggests that speech sounds strongly activate the motor cortex e.g., Flöel et al. (2003). However, other evidence suggests that the motor cortex might be also activated by non speech sounds (Watkins et al., 2003; Alibiglou and Mackinnon, 2012) so we also included a condition in which the auditory stimulus matched the amplitude envelope of the speech stimulus but consisted entirely of white noise (Pulvermüller et al., 2006). This signal-correlated noise (SCN) stimulus was created using Praat (Boersma and Weenink, 2013). Time and frequency domain comparisons of the two signals are displayed in Figure 1.

The order of all seven auditory-stimulation/TMS pairs and stimulus types (speech or SCN) was randomly intermingled and presented with an intertrial interval (ITI) that randomly varied between 4000 and 5000 ms in two blocks such that the total number of stimuli per condition was 16. The total number of trials was hence 128 (16 baseline trials + 7 × 16 conditioned trials). The duration of the experiment was approximately 25 min.

### Data processing

Offline MEP analysis was conducted using a custom MATLAB (The Mathworks, USA) script. The average MEP amplitude calculated for each sound type and auditory-stimulation/TMS pair was expressed as a function of the average pre MEP (baseline).

### Statistical analysis

A repeated measures ANOVA with the factors delay (auditory-stimulus/TMS interval) and condition (speech or SCN) was performed on the averaged MEPs. A two-tailed, one-sample *t*-test was then used to determine the time points at which the conditioned MEPs differed significantly from baseline using an α-value of 0.05.

## Experiment B—auditorimotor PAS

### Subjects

Experiment B was performed on 10 healthy right-handed volunteers (8 males), aged 18–31 years (mean 24.5 ± 3.3 years) without any prior neurological medical history. Written informed consent was obtained from each subject before participation in the study.

### Experimental procedure

The procedure used in Experiment B was similar to the one used in Experiment A. The main difference was that a single auditory stimulus/TMS interval (100 ms) was used during the PAS induction period in Experiment B. As no difference in MEP facilitation between the speech and SCN stimulus conditions was found in Experiment A we arbitrarily chose to use only the speech stimulus in Experiment B. PAS induction following baseline MEP recording consisted of a total of 200 auditory stimulus/TMS pairs applied with a 4000–5000 ms random interval between each pair. A 2 min pause in stimulation after 100 pairs were applied was included. The total duration of the experiment was approximately 27 min (introduction: 10 min, part one: 7.5 min, pause: 2 min, part two: 7.5 min).

### Statistical analysis

A two-tailed, one-sample *t*-test was used to determine significant differences between pre-MEPs (baseline), post-MEPs and post15-MEPs using an α-value of 0.05.

## Results

Mean (± SEM) MEP threshold in Experiment A was 45.5 ± 2.1% of stimulator output and 46.6 ± 2.4% in Experiment B.

**Figure 2:**
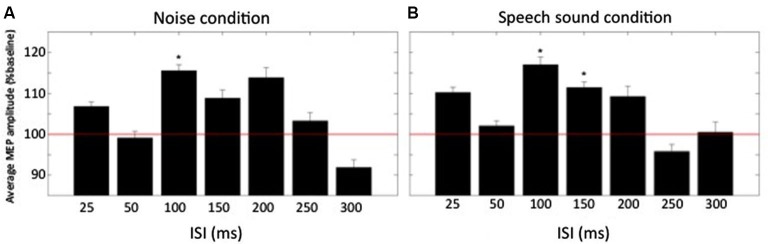
**Normalized averaged MEP amplitudes (+SEM) at different times relative to the conditioning auditory stimulus**. **(A)** MEP amplitudes for the condition “noise” for all ISIs (*n* = 12). **(B)** MEP amplitudes for the condition “speech sound”. Baseline is represented by the red horizontal line. * denotes average amplitude significantly different from baseline (*p* < 0.05).

Results from Experiment A are shown in Figure 2. A repeated measures ANOVA showed that there was a significant effect of delay on the size of the MEP *F*_(6,66)_ = 2.3, *p* = 0.045. There was no significant effect of condition nor significant interaction between delay and condition. Within condition comparison of mean normalized MEPs to baseline by means of a two-tailed one-sample *t*-test revealed that in the noise condition, MEPs were significantly increased above baseline for one ISI: 100 ms (115.5 ± 5.2% of baseline, *t*_(11)_ = 3.0, *p* = 0.012). For the speech sound condition two ISIs had MEPs that were significantly increased above baseline: ISI = 100 ms (117.0 ± 6.5% of baseline, *t*_(11)_ = 2.6, *p* = 0.023) and ISI = 150 ms (111.4 ± 4.7% of baseline, *t*_(11)_ = 2.4, *p* = 0.035).

**Figure 3:**
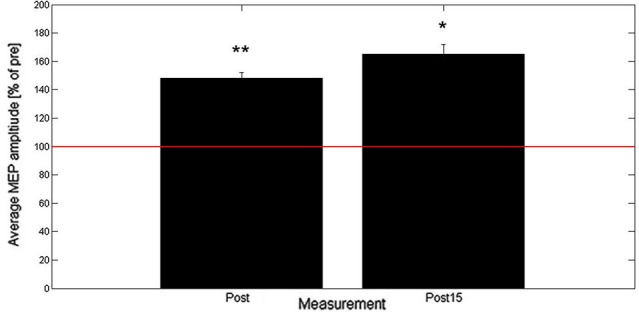
**Averaged post- and post15-MEP-amplitudes (+SEM) as percentage of baseline (*n***= 10)**.** Baseline is represented by the red horizontal line. * denotes average amplitude significantly different from baseline (* < 0.05, ** < 0.01).

Results from Experiment B show that across all subjects the averaged MEP peak-to-peak amplitude increased to 148% (post) and 165% (post15) of baseline as shown in Figure 3. Two-tailed one-sample *t*-tests showed a significant increase in normalized MEP peak-to-peak amplitude for post (*t*_(9)_ = 3.8, *p* = 0.004) and post15 (*t*_(9)_ = 2.9, *p* = 0.018). Comparison between post and post15 by means of a paired *t*-test revealed no significant difference (*t*_(9)_ = 1.06, *p* = 0.32).

## Discussion

The current study demonstrates for the first time that long-lasting motor cortical plasticity can be induced by an auditorimotor PAS paradigm. This result is significant because it not only provides a new method for investigating auditorimotor integration, but importantly, also a method to directly probe the brain’s capacity for auditorimotor plasticity.

We utilized a two-stage approach in developing this PAS paradigm. First, we identified the optimal ISI for eliciting an enhanced MEP response compared to baseline. This paradigm follows the empirical approach developed by Mrachacz-Kersting et al. (2007) to investigate PAS induced plasticity in the cortical representation of tibialis anterior. The optimal interval we found fits well with the temporal structure of the auditory N1 to speech sounds which peaks 100 ms after stimulus onset e.g., Liotti et al. (2010), and agrees with the TMS findings of Fadiga et al. (2002) and those of Roy et al. (2008), who found “phonological motor resonance” was present at 100 ms after their target speech sound stimulus onsets. In both studies the authors applied TMS to the tongue motor representation following the presentation of pseudo-words containing double consonants. The MEP response that they recorded in the tongue peaked in amplitude when the auditory stimulus to TMS interval was 100 ms.

While we used a speech stimulus in these experiments, the lack of difference between the response to the speech stimulus and SCN found in Experiment A suggests that under the experimental conditions we have imposed, i.e., a repetitive presentation of a speech sound without the requirement for engagement on the part of the subject, the stimulus may not be processed as speech *per se* and should rather be considered a non-specific acoustic stimulus. This fact may explain why our results differ in part to those of Watkins et al. (2003) and Murakami et al. (2011) whose findings suggest that auditory-induced motor modulations related to speech listening are confined to the cortical representations of those muscles involved in articulation. Indeed, there is now a significant body of evidence to support the somatotopic arrangement of speech gesture perception (Fadiga et al., 2002; Roy et al., 2008; D’Ausilio et al., 2009, 2011; Möttönen and Watkins, 2009; Sato et al., 2010) but such findings do not necessarily rule out the non-specific motor activations in response to both speech and non-speech acoustic stimuli that have been documented using both TMS and other methods (Flöel et al., 2003; Alibiglou and Mackinnon, 2012; Fujioka et al., 2012).

The current study shows that repeated pairing of an acoustic stimulus with a TMS pulse to the motor cortex representation of the hand leads to a rapidly-evolving, long-lasting increase in cortical excitability. This effect was induced with an ISI of 100 ms, a time interval that corresponded to the point of peak enhancement in the acoustic stimulus-conditioned MEP. Given that this ISI was converged upon using a method that used discrete intervals with a minimum step of 50 ms, it is expected that this PAS technique could be refined further by re-examining the optimal sound-to-TMS interval using smaller time steps (i.e., less than 25 ms) centered around 100 ms. Moreover, using auditory evoked potentials to discover individualized N1 latencies, and then using these as the basis for the PAS ISI would likely refine the technique further. Since we were able to find a significant PAS effect in this proof of concept study, we posit that auditorimotor PAS is a robust effect that will provide a powerful tool for studying auditorimotor plasticity in the future.

Auditorimotor plasticity i.e., the capacity for strengthening of auditorimotor connections within the brain is essential for the acquisition of speech and the learning of musical competence. For this reason, techniques that can probe the brain’s capacity for auditorimotor plasticity provide the opportunity to investigate some of the hypothesized mechanisms of conditions such as stuttering and specific language impairment (SLI) in which disordered motor learning has been documented (Namasivayam and van Lieshout, 2008; Mayor-Dubois et al., 2014). Both of those conditions have been associated with disordered sensorimotor integration (Hill, 2001; Neef et al., 2011; Cai et al., 2012, 2014) and, in the case of SLI, with disordered auditorimotor plasticity (Kurt et al., 2012). Additionally, disorders such as schizophrenia and tinnitus have been associated with disrupted auditorimotor connections (Cacace, 2003; Ford et al., 2005; Langguth et al., 2005) and synaptic plasticity (Møller, 2003; Stephan et al., 2009); the technique described herein is therefore a novel means to assess these associations. Beyond mechanistic investigation of disorders, associative stimulation using TMS has also been proposed as a therapeutic modality (Uy et al., 2003; Jayaram and Stinear, 2008; Michou et al., 2013). If it is established that disorders such as those described above involve a form of auditorimotor disconnection, then auditorimotor PAS could be used as a novel adjuvant therapy to assist in the re/establishment of appropriate sensorimotor mappings.

## Conflict of interest statement

The authors declare that the research was conducted in the absence of any commercial or financial relationships that could be construed as a potential conflict of interest.
